# From Our Correspondents

**Published:** 1986-02

**Authors:** 


### From Our Correspondents

### Disaster Day at Taunton

What does a District General Hospital histopathologist do when he breezes into work one Monday morning to find 500 mutilated corpses awaiting his attention? That, you may reply, is statistically unlikely, but in 1986 it is a spectre constantly lurking around the mortuary corner.

Consider, for example, what would have happened if the Air India airliner which crashed off Southern Ireland last summer had been as far ahead of schedule as its sister aircraft in which (shortly after landing at Tokyo the same day) a bomb blew up and murdered two baggage handlers. There would have been a devastating explosion somewhere over South West England, with 329 fragmented and burned bodies scattered over a large area, perhaps with many more victims on the ground. Coping with the identification, autopsies, toxicology and other pathological aspects of the disaster investigation would have occupied two West Country pathologists full time for at least 2 weeks. It would have left them with enough loose ends and court work to fill a book, not to mention irate surgeons and physicians demanding their routine histology, cytology and post mortem reports.

This kind of grim calculation was behind a meeting of the Far West Histopathology Group held at Musgrove Park Hospital, Taunton in December. Its members are a professionally tight-knit group of pathologists whose practices stretch from Sedgemoor to Land's End. Remoteness from the temples of Academe makes the spirit of self-help beat stronger: the group has already, through the work of Dr Anthea Sherwood at Torbay Hospital, pioneered a scheme of microscopic self-audit which may be adopted as a blue print for other schemes by the DHSS.

On Friday, 13 December, however, disasters and not diagnosis were the main agenda. The date was not well chosen and the ever present risk of air disaster was tragically emphasised 24 hours earlier by the Gander crash which brought an unprecedentedly bad year for civilian air fatalities (nearly 2000 of them) more or less to a close.

Yet air crashes, as one speaker remarked, are only one source of mass death. The South West peninsula has its share of motorway madness, crowded shipping lanes, high speed rail, dock yards, ordnance factories and nuclear energy plants. A paranoid pathologist west of Avon does not lack horror spots to keep him awake at night. The last significant disaster was the Taunton train fire with 13 dead in July 1978, and the laws of probability state that something nasty will strike the South West again one of these fine days.

So how *does* your beleagured pathologist colleague cope with 500 dead on a Monday morning? The strict but naive answer is that he does not have to; the responsibility in all forms of unnatural or violent death lies with H M Coroner, who can make whatever arrangements he wishes or is able to make. That might include calling in the Home Office forensic pathologist, or the local university staff (if there is one), or the RAF aviation pathology team. In real life these last resorts are often not available and the local man may well be 'it' by himself, with a little help from his friends.

It was this possibility that the speaker at the Taunton Disaster Day, Group Captain Tony Balfour FRCPath addressed. Group Captain Balfour heads the RAF Department of Aviation Pathology and Forensic Medicine at the RAF Institute of Pathology, Halton, in Buckinghamshire. His unit has unique expertise within the UK and most of the jetsetting world in the investigation of over 1,000 military and civil air disasters, many of them involving multiple fatalities. Although aviation disasters, as I have pointed out, are only one form of mass death, the medico-legal and logistic principles involved make them a very useful study for land based and maritime accidents. Equally important, aviation disasters over the last 30 years have demonstrated time and again the vital contribution that the pathologist can make toward accident prevention and increasing accident survivability. It is this contribution which transforms a generally unpleasant experience into a worthwhile and challenging professional task.

The simple message derived from the RAF experience is that mass disasters may concentrate the mind wonderfully, but they are a bad time for pure extemporization by pathologists, or anyone else. Therefore it is reassuring to know that whereas your hospital disaster plan usually allow dead victims a brief para at the bottom of the last page, the police do better. The police occupy a pivotal role in the aftermath of mass disasters. They control the site, provide the communications, co-ordinate the efforts of fire, ambulance, military and voluntary services, authorise the movement and release of bodies, liaise with the press and relatives and co-operate in many aspects of identification. Every police force in the country has a printed Force Main Incident Guide which defines clearly the responsibilities and procedures necessary, often with regard to a particular scenario like an explosion at a specified nuclear power station. If the topic interests you further in a professional and non-ghoulish way, a talk with your local Chief Superintendent or his nominated Staff Officer and a request to look over the local Main Incident Guide is a good starting point.

In between some of the most frightening slides of the year (the kind that bring 3rd MB students flocking in triple figures) Group Captain Balfour offered some guidance on how the harrassed pathologist guards himself from being totally swamped. His recommendations were often specialised and I do not wish to spoil your next meal. Basically they urged a careful systematic procedure, starting at the accident site and continuing at the mortuary. It relies heavily on team co-operation (with police, forensic odontologist, dentists, non-medical investigators and undertakers) and keeping all the bodies in one location until all identification had been satisfactorily achieved. There is no room for the brilliant but tearaway Dr Quincy in this specialty.

Finally the speaker described his ideal mortuary for mass disasters. The hospital or normal public mortuary is the last place you select. In no time the access roads are blocked by police cars, hearses and TV crews; your doors are besieged by sightseers and souvenir hunters who spring out of the ground and your telephone lines by relatives, press men and cranks. In addition your hospital routine is totally disrupted and there is no room in the fridges for ordinary hospital cases. The ideal location is a remote, roomy, windowless building, miles from anywhere which can be firmly secured against media intruders and grieving relatives. It needs arc lamps, trestle tables, pinboards and sawdust, all of which can be assembled in large quantities. Running water is a bonus not a necessity and if you must have refrigerated body storage, refrigerated vans can usually be obtained from the wholesale foods industry on a very confidential basis of course.

A. E. Adam

**New M.R.I. scanner to be sited at Frenchay**

Further to the recent contribution from Frenchay Hospital about Magnetic Resonance Imaging (M.R.I.), there is now some unexpected but momentous news to report, which by the time this column is likely to be published, is bound to be widely known, but nevertheless should be written down for the record if nothing else.

During the month of November, some behind-scenes discussions were taking place which culminated in the John James Trust decision to purchase outright an M.R.I. scanner for the Bristol hospitals, at close on one million pounds. This is the largest single medical gift, ever to be donated by the Trust, and is a wonderful donation by a Trust that already has an outstanding record of supporting hospitals, hospices, schools and old age pensioners, and other in the years past. In view of the availability of ground, and the closeness to the motorways to enable a Regional service to be provided, Frenchay Hospital was chosen as the site for installation, and the Frenchay Health Authority responded with alacrity in offering a splendid site on the north side of the hospital close to the conservation area. In fact a building plan has already been agreed, within three weeks of the announcement, and it is hoped to commence building on March 1st 1986.

In the meantime, the scanner is being built, and as this normally takes six months, the building should be sufficiently advanced to receive the machine in the coming summer, with an opening date visualised in the autumn. It is planned that all three Bristol hospitals will share time on the machine with a permanent staff of technicians, and back-up staff, to provide the continuity. The revenue will thus be shared between the three Bristol Health Authorities, which will lessen the considerable financial burden involved.

However, all the hurdles are not yet crossed and much remains to be done. Most important of all, we have been challenged, as Bristolians, by John James to raise the money needed for the building and for the first few years revenue. We have of course accepted this challenge with enthusiasm and determination for if one Trust can produce such a marvellous gift, surely the local population in the south-west can rise to the occasion, and produce the required half a million pounds to complete the project. An Appeals Committee has therefore been set up, and already several schemes have been planned including television programmes and raffles. If any reader of this column can assist, by suggesting other methods, or putting us in contact with individuals or business firms who might be interested in donating towards this Fund, please let us know. The title 'The Bristol M.R.I. Scanner Fund' needs to be top of the list for several months to come. The Fund is a registered charity (No. 246724). Please help all you can!

J. L. G. Thomson

**Care of the elderly**

In my last article I mentioned the Diploma in Geriatric Medicine, and readers might like to know that about 120 candidates sat the first examination on November 25th. Although the majority of the candidates were younger doctors, the Diploma attracted a fair number of established general practitioners. Such a response justifies its establishment, despite the doubts raised in the minds of a few when the concept was initially discussed.

The British Geriatric Society is going from strength to strength. Its recent annual meeting in London attracted the largest audience ever, with over 400 people in attendance. During the meeting there was debate, both official and unofficial, over the role of the journal, Age & Ageing, and its relationship to the Society. I for one was surprised to learn that it was not officially the Society's journal. It is heartening to note, however, that all papers submitted for publication will now be vetted by referees, as is usually the case in most respectable journals. The Society has also established a Scientific Committee to which a representative of the South West was successfully elected. I shall watch with interest as the objectives and role of this committee expand.

The Bristol Dementia Research Programme is getting under way with a variety of projects, clinical, neuropathological and neurochemical, delving into different aspects of dementia in the elderly, especially of the Alzheimer type. The author of these few paragraphs would be pleased to hear from anyone who is interested in collaborating – our special need at the moment is for postmortem material from cases that have been carefully assessed clinically.

Finally, may I wish Dr Brandon Lush, albeit a little belatedly as far as these columns are concerned, a very happy and active retirement. It is several months since he left us at Frenchay Hospital, although we have noted with pleasure his presence at local meetings.

G. K. Wilcock

**Clinicosis**

By this, I mean unnecessary attendance at clinics, whether these be for healthy babies to be smiled at, for post mastectomy patients to be palpated for years on end, for chest patients to have x-rays and sedimentation rates years after they had tuberculosis, or for elderly folk to be screened by unrewarding blood tests and electrocardiograms. The motives for doctors to practise clinicosis vary from a desire to boost their own ego by seeing grateful patients, to being over-cautious and obsessional about not missing something, to boosting their attendance figures and self-importance. There may be political motives such as preserving a clinic, and persuading management of the need for more staff or resources.

The problem is compounded by the desire of some patients to remain 'under the hospital' where they gain spurious confidence from seeing a variety of junior staff over the years. I have had some patients whom it is difficult to wean off anticoagulants, long after I have considered the warfarin should be stopped. Others insist on being seen regularly for diabetes or hypertension as they only get repeat prescriptions from the family doctor. There is of course a genuine need for regular attendance where eye examinations, blood or other tests need to be done, though fortunately an increasing number of health centres are now monitoring these types of patient well, and getting the opticians to check their eyes, on the model described by Dr. Burns Cox recently in the British Medical Journal.

I feel there is a strong case for prolonged follow-up of some conditions such as epilepsy, where expert knowledge of modern drugs and their complications is available, together with facilities for seeing a social worker in this disorder which can have profound behavioural problems. This case is reinforced if there is an occasional need for repeated blood, electrophysiological or radiological monitoring. There is an incontrovertible argument for clinicosis if a doctor is involved in a well planned prospective trial of a new procedure or medication. Follow-up of such patients in the hurly burly of a general clinic is never satisfactory; it is always better to start a special clinic or allocate an hour or so at the end of the routine out-patients. I feel there is also ample justification for an occasional review of certain disorders for the purpose of teaching undergraduates or junior staff, for example some cardiac murmurs, orthopaedic anomalies, enlarged organs and unusual skins. A further example of the value of follow-up is to study the natural history of unusual conditions – an example being spontaneous closure of ventricular septal defects. By such studies, advances in our knowledge are made.

Nevertheless, throughout the kingdom many clinics are crowded out by needless old patients. The only way we can reduce the burden is by all hospital doctors questioning the need for a repeat attendance, and by family practitioners taking over the long term surveillance of many maladies. This should allow some shortening of the waiting list for new out-patients which in some disciplines is a disgrace to the National Health Service.

H. G. Mather

**Security of tenure: stability?**

How secure are any of us? The issue for academics has been aired recently in Letters to the Editor, columnists' gossip and even in the stern leaders of *The Times*. Non-professorial, but senior, clinical academics in the University of Bristol used to be issued merely with a 'Letter of Appointment'. I found my own Letter by chance the other week during yet another move from more room to less room. Financial Contraction it appears but, no Contract. There was in the 'Letter' merely reference to an apparently changeable set of regulations approved by the University Council. Hey ho!

Is such absence of Contract universal, one wonders innocently. The very next thing that fell out of that same drawer was what I had thought was a 'Contract' with a publishing firm. No such stability. I found that it was just headed as a 'Memorandum of Agreement'.

The world is clearly falling apart. Is no one now willing to make a Contract? Are the times really so unstable? Does Contract Bridge Survive or is that, too, an old-fashioned term? Do proper consultant contracts exist, or are they in danger of being rewritten as temporary 'Agreements' with the current Minister of Health. New version next election.

J. D. Davies

**From Ham Green Hospital**

Dr Denis Wright reports that he is well and back at work again after an 'escapade' at Frenchay. He also reports that Dr Stewart Glover has been awarded the Gilliland Travelling Fellowship for 1986, and will visit the Lassa Fever Research Unit at the Nixon Memorial Hospital in Sierra Leone.

He also offers us the following quotation as an apt commentary on the direction of the NHS since 1974 –

'We trained hard – but it seemed that every time we were beginning to form into teams, we would be reorganised. I was to learn later in life that we tend to meet any new situation by reorganising, and a wonderful method it can be for creating the illusion of progress, while producing confusion, inefficiency and demoralisation'.

Gaius Petronius 66 AD.

